# Brains vs Brawn: Relative brain size is sexually dimorphic amongst weapon bearing ruminants

**DOI:** 10.21203/rs.3.rs-3143852/v1

**Published:** 2023-07-21

**Authors:** Nicole Lopez, Jonathon Moore Tupas, Theodore Stankowich

**Affiliations:** 1 Department of Biological Sciences, California State University, 1250 Bellflower Blvd., Long Beach, CA 90840; 2 Division of Biological Sciences, University of Montana, 32 Campus Drive, Missoula, MT, 59812

## Abstract

Here, we investigate the relationship between relative brain size and sexual weapons in ruminants. In most cases, sexual weaponry is heavily male-biased, and costs resulting from growing, maintaining, or wielding weapons will be suffered primarily by males. We used comparative phylogenetic analyses to test whether increased investment in sexual weapon size (tusks, antlers, and horns) across four families (Tragulidae, Moschidae, Cervidae, and Bovidae) was associated with decrease in relative brain size, and whether the difference in weapon investment relative to conspecific females led to sexual differences in relative brain size. We found no relationship between relative brain size and relative weapon size within males or females, but when we compared males directly to conspecific females, we found that as males possessed larger weaponry, they had smaller brain sizes, regardless of weapon type. Our finding suggest male investment in some types of elaborate weapons could be related to male reduction in larger brains.

## Introduction

Natural selection favoring greater cognitive ability is hypothesized to explain the evolution of large brain sizes in many bird and mammal species (Eisenberg & Wilson, 1978; Iwaniuk et al., 2001; Jerison, 1973; Lefebvre et al., 2004; Reader & Laland, 2002; Sol et al., 2005a; Tsuboi et al., 2020). The “expensive brain” hypothesis predicts that energy spent to develop and maintain large brains will result in the diminishment of other expensive physiological functions including reproductive rates (Isler & van Schaik, 2006) and morphological structures like gut (Kotrschal et al., 2013) and testes size (Lemaitre et al., 2009). A recent study in mammals showed that significant investments in morphological antipredator defenses (e.g., spines/quills, dermal armor, noxious sprays) were associated with reductions in relative brain size, suggesting that selection favoring costly morphological defenses can overwhelm selection favoring advanced cognitive abilities, especially in dangerously exposed environments (Stankowich & Romero, 2017). Given that intrasexual selection strongly favors elaborate sexual weapons in male ruminant mammals (tusks, horns, antlers) and these can be costly to grow, maintain, and carry around (Festa-Bianchet et al., 2004; Landete-Castillejos et al., 2019; Loe et al., 2019; Moen et al., 1999; Mysterud et al., 2005), we investigated whether investment into such expensive structures might also have resulted in an negative evolutionary relationship with relative brain size between males and females of the same species (i.e., when males evolve to invest more in their weapon, does their brain size decrease relative to females of the same species?).

Males of many species expend tremendous energy growing elaborate, often heavy sexual weapons used to signal fighting strength and to fight with other males for access to reproductive females (Emlen, 2008; Landete-Castillejos et al., 2019). The sexual weapons of ruminant mammals are particularly well studied and vary considerably in size, shape, weight, growth patterns, and use in battle (Caro et al., 2003; Davis et al., 2011). For example, large upper canines, tusks, are only used in sexual combat (Barrette, 1977; Dubost & Terrade, 1970; Wilson & Mittermeier, 2011) and are found exclusively on males in three extant deer families: Tragulidae (mouse deer; Cabrera & Stankowich, 2018; Wilson & Mittermeier, 2011), Moschidae (musk deer; Fennessy, 1984; Wilson & Mittermeier, 2011), and some Cervidae (Chinese water deer (*Hydropotes inermis*), muntjacs (*Muntiacus* spp.), and tufted deer (Elaphodus; Wilson & Mittermeier, 2011). Antlers are found almost exclusively in the males of all cervids (true deer) except the Chinese water deer, and both male and female caribou (*Rangifer tarandus*) bear antlers. Muntjacs and tufted deer possess both tusks and antlers, where antlers are used in dominance displays before combat with tusks (Barrette, 1977). Finally, true horns are the sole sexual weapons in the Bovidae (e.g., antelope, goats, bovines) and have evolved into many different shapes and sizes based on fighting style and are also used both in combat and/or visual intrasexual male contests (Caro et al., 2003); many female bovids also grow horns, although they are usually shorter and weaker than the males’ horns of their species (Stankowich & Caro, 2009).

While antlers are deciduous (i.e., shed and regrown annually), tusks and horns are permanent. For most cervid and bovid species, antler and horn sizes increase quickly during the first few years until males reach full adult size; from that point weapons increase in size gradually with age (Fennessy, 1984). Within species, static samples of adult antlers, horns, and tusks scale disproportionately steeply (i.e., hyperallometrically) with body size (Lopez & Stankowich, 2023), resulting in some cases of extreme weapon sizes in the largest individual males. This pattern of weapon expression is predicted when costs of weapon production outweigh the benefits of large weapons for poor condition or relatively small individuals (Emlen, 2008; Emlen et al., 2012; Kodric-Brown et al., 2006; Nur & Hasson, 1984), which suggests that investment in sexual weapons in these species could limit relative investment in other growing structures.

Because sexual weapons in the majority of ungulates are male biased, we hypothesized a negative relationship between weapons and brains, where increased investment in weaponry leads to decreased investment in brain size. We predicted this effect within each sex, but we also predicted that as sexual dimorphism in relative weapon investment increases in a species (i.e., as males evolve larger weapons relative to females of their species), male brain size will decrease relative to females of the same species. We tested for relationships between sexual weaponry and relative brain size in ruminant artiodactyls by measuring weapon length (canine, antler, or horn), skull length, and endocranial volume from male and female skulls of 8 tusk-bearing species across three families (Tragulidae, Moschidae, and Cervidae), 13 antler-bearing cervid species, and 11 horn-bearing bovid species (Fig. 1A). We calculated sex-specific measures of relative brain and weapon size and used comparative phylogenetic analyses to test our predictions.

## Methods

### Data Collection

In this study, we tested the effects of sexual weaponry on relative brain size in ungulates bearing three different weapon types: horns, antlers and tusks. Four hundred and thirteen specimens (*N_horn_ = 113, N_antler_ = 171, N_tusk_ = 131*) from 29 species were measured at the following museums: National Museum of Natural History (NMNH), Natural History Museum of Los Angeles County (LACM), CSULB Collections (CSULB), American Museum of Natural History (AMNH), California Academy of Sciences (CAS), and the Field Museum of Natural History (FMNH). We took measurements on both adult male and female specimens, and only included species in our analyses where we had complete measurements from at least three individuals of each sex (one exception, *Rangifer tarandus,* only 2 females measured). While our final sample was 29 species, we want to note how difficult it is to find at least 3-5 fully intact male and 3-5 fully intact female skulls (that include at least one weapon and a cranium that isn’t broken to measure volume) of ungulate species in natural history museums, and the time it takes to take these measurements. We feel that the fact that we were able to detect a significant effect despite having only 29 species (and fewer in the separate weapon tests) suggests a strong negative relationship and a more conservative approach.

We collected the following cranial measurements. Skull length (mm) was measured from the anterior tip of the premaxilla to the most posterior point of the skull (typically the occipital crest or occipital condyles) (Fig. S1a, Online Resource). Skull width (mm) was measured transversely from one zygomatic arch to the other (Fig. S1a, Online Resource) at the greatest width of the skull. Skull height (mm) was measured from the lowest point on the squamosal at the back of the skull to the highest point on the dorsal midline of the cranium (Fig. S1b, Online Resource), not including the antlers/horns or pedicels. Endocranial volume was measured by filling the skull with 3mm glass beads (smaller skulls) or 6x9mm plastic beads (larger skulls) through the foramen magnum, and then measuring the volume of beads (mL) in a graduated cylinder. Due to the curved nature of tusks, we collected two measurements: (1) from the most mesial point on the buccal surface where the tooth emerges from the skull to the tooth tip, and (2) from the most distal point on the buccal surface where the tooth emerges from the skull (Fig S1b, Online Resource). Then we took an average of both values from each complete, unbroken tusk and used the value from the longest tusk as our weapon length (mm). The cranial weaponry data were collected by measuring the curl of the antler or horn using a flexible measuring tape (mm). Antler curl was measured as the greatest length from the posterior lateral base, along the outer curved surface, to the tip of the antler (Fig. S2, Online Resource). Horn curl was measured as the average of the lengths of the maximum and minimum curvature ridges of the largest horn on each skull (Fig. S3a; S3b, Online Resource). We then used the average of the two lengths of the largest horn on each skull for horn length.

All skull and tusk measurements were collected using digital calipers to the nearest 0.01mm then converted into centimeters (cm), and all antler and horn measurements were collected using a flexible measuring tape to the nearest 1cm. As both weapon length and endocranial volume were required on all skulls, only intact skulls were used, and any broken skull dimensions or teeth were not measured. From these raw measurements, we calculated skull volume (SkV) (mm^3^) as the product of Skull Length x Skull Width x Skull Height and brain mass (BM) (g) as the product of Brain Volume (mL) x1.036 (g/mL; Stephan et al., 1981).

From these baseline measurements, we used traditional methods of calculating relative brain size to generate two variables, Weapon Quotient (WQ), a new measure, and traditional Encephalization Quotient (EQ) (Boddy et al., 2012; Jerison, 1973). Skull length (cm) was used as a representation of body size over body mass since it was individually measured for each specimen and body mass would be a less accurate species average. First, we ran separate linear regressions of log_10_-transformed male average weapon lengths (WL) for all species averages (tusks, antlers, and horns combined): WL (cm) vs Skull Length (cm) (Skl). Next, we ran linear regressions of log_10_-transformed species average brain mass (BrM) versus body size for all species: BrM (g) vs SkL (cm^3^). Lastly, we phylogenetically corrected our results by using the function ‘pgls’ (Orme, 2013) resulting in a different set of parameters and only these corrected values were used in the following calculations (Table 1)

Next, we used the resulting corrected ß (slope) and b (intercept) estimates to calculate the predicted brain masses and weapon lengths for each individual specimen based on their individual skull lengths: BrM_*i(predicted)*_ = 10^b(BrMvsSkL)^ × SkL_*i*_^ß(BrMvsSkL)^; WL*_i(predicted)_* = 10^b(WLvsSkV)^ × SkV_*i*_^ß(WLvsSkV)^. EQ_*i*_ for each individual skull was calculated as BrM*_i(measured)_*/BrM_*i(predicted)*_, where an EQ above 1.0 would represent a relatively large brain and an EQ below 1.0 would be a relatively small brain. Similarly, WQ*_i_* for each individual skull would be calculated as WL_*i(measured)*_/WL_*i(predicted)*_ and be interpreted the same way relative to a value of 1.0. Antler WQ was automatically set to zero for almost all female cervids, with the exception for antlered female caribou (*Rangifer tarandus*).

We then calculated the average EQ and WQ for the male and female specimens for each species, resulting in average EQ_♂_, WQ_♂_, EQ_♀_, and WQ_♀_ for each species based on either skull length or body mass (8 total measures for each species). Next, we calculated the difference between EQ_♂_ and EQ_♀_ (ΔEQ) and the difference between WQ_♂_ and WQ_♀_ (ΔWQ) for each species. A result of ΔEQ below 0 indicates that females have relatively larger brains than males in those species. Since females of antlered species almost exclusively had WQ_♀_=0, ΔWQ = WQ_♂_ with the exception for antlered female caribou (*Rangifer tarandus*). We used these species averages to determine whether there is a sexually dimorphic relationship between males and females in relative brain investment, and whether males suffer a physiological trade-off between weapon length and relative brain investment.

For the following species only*: Elaphodus cephalophus, Muntiacus reevesi, Muntiacus muntjak*; WQ is a sum value of WQ for tusk and antler measurements (WQ = WQ_tusk_ + WQ_antler_) because these species bear both weapons and invest differently in each type respectively. This method allows us to generate a total ‘weapon’ investment. These species are categorized and represented as antler bearing species in our final analyses since previous work supports antlers scale positively allometrically while tusks scale isometrically suggesting greater in investment in antlers over tusks (Lopez & Stankowich, 2023). In addition, we ran separate analyses amongst each weapon type individually (*Horns=11, Antlers=13, Tusks=8*). WQ was weapon specific (tusk WQ or antler WQ) for *Elaphodus cephalophus, Muntiacus reevesi, Muntiacus muntjak.* All additional analyses can be found in our supplemental data (Table S2; Figure S6; S7).

### Statistical Analysis

We ran a phylogenetic generalized least squares (Martins & Hansen, 1997), ‘pgls’ tests using the ‘ape’ (Paradis 2019) the ‘caper’ package (Orme, 2013) in R (Team, 2020) across a consensus tree pruned from the Upham et al. (2019) DNA-based consensus mammal-wide tree (N_tree_= 29; Fig. 1A). We tested the relationship between EQ and WQ amongst males solely, female solely, and for the sexually dimorphic relationship (M-F ΔEQ vs ΔWQ) and included weapon type as a factor (Table 3). In addition, we ran phylogenetically corrected tests for interaction effects on WQ or ΔWQ. All additional results can be found in our Online Supplement. We set our significance level at α = 0.05 and calculated phylogenetic signal for each test using maximum-likelihood estimations of lambda (λ) derived from the PGLS tests.

All additional supplemental analyses (each weapon type tested individually) were ran using ‘pgls’ using the ‘ape’ (Paradis, 2019) and ‘caper’ package (Orme, 2013) in R (Team, 2020) across a *weapon specific* consensus trees pruned from the Upham et al. (2019) DNA-based consensus mammal-wide tree (i.e., Horn Tree, Antler Tree, Tusks Tree).

## Results

Overall, weapon size scales hyperallometrically with body size (as estimated by skull size) suggesting that as individuals (male biased) grow larger, they grow disproportionately larger weapons (Table 2; Fig. S3), similar to findings in other studies (Gould, 1974; Lopez & Stankowich, 2023; Plard et al., 2010). Similarly, brain mass (g) scaled hypoallometrically with body size (as estimated by skull size; Table 2; Fig. S4), similar to findings in other studies (Boddy et al., 2012; Heldstab et al., 2016; Huang et al., 2020). While our results provided further support for these previously established relationships, we next examined the relationship between relative investment in brain size and relative investment in weapon size.

We calculated sex-specific measures of relative investments in brain size (encephalization quotient: EQ) and weapon size (weapons quotient: WQ) by correcting for body size (as estimated by skull size): EQ and WQ scores above 1 indicate greater than expected investment in these structures and scores below 1 indicate smaller investments relative to body size. For males, we did not find any effect of investment in weaponry on brain size (Table 3; Fig. 1B), although male antlered species had significantly greater investment in their brains than tusked and horned species (p < 0.05; Figure 1B; Table 3). We did not find any effect of investment in weaponry on brain size in females (Table 3; Fig. 1C), although, again, female antlered species had significantly greater investment in their brains than tusked species (p < 0.001).

We found a significant negative relationship between the degrees of sexual dimorphism in relative brain size (ΔEQ) and relative weapon size (ΔWQ; Table 3, Fig. 1D), whereas males evolve to invest proportionally more in weapons than females of their species, they evolve to invest proportionally less in brain size (p = 0.014). This result supports that investment in relative brain size is sexually dimorphic and likely influenced by the presence of exaggerated sexual weapons in these male ungulates. We also found that the difference between male and female relative brain size investment was greater in antlered species than horned species (p = 0.049; Figure 1D; Table 3)

Phylogenetic signal in the analyses was either completely absent (λ = 0.000) or very strong (λ near or equal to 1; Tables 2 & 3, S1), suggesting great variation in the degree to which shared ancestry explains variation in relative brain and weapon size. We did not find any evidence of an interaction effect of WQ on EQ in any analyses, so the interaction term was dropped from all final models (Table S1). In addition, we ran separate analyses amongst each weapon type individually (*Horns=11, Antlers=13, Tusks=8).* For all groups, we found similar insignificant results between male WQ and male EQ, except amongst tusked species, we found a significant negative relationship between male WQ and EQ and female WQ and EQ. Lastly, for our sexual dimorphic analysis, we found similar results in our tusked and horned groups, with significant negative relationships between M-F EQ and M-F WQ. However, we did not find any relationship amongst our antlered group. All additional analyses can be found in our supplemental data (Table S2; Figure S6; S7).

## Discussion

Our data support the hypothesis that increased investment into male weapon size is associated with a sexual dimorphic investment in relative brain size. Across twenty-nine species and three weapon types (horns, tusks, antlers), as males evolved to invest relatively more than conspecific females in building larger weapons, they invested relatively less than conspecific females in building larger brains. Past studies support physiological and behavioral tradeoffs when males possess large, exaggerated weapons (e.g., reduced limbs (Emlen, 2001; Simmons & Tomkins, 1996), reduced efforts in nuptial gift giving (Liu et al., 2015), survival rate (Douhard et al., 2020; Garratt et al., 2015) or increased grooming time (Allen & Levinton, 2007; McCullough et al., 2020)) but this is the first study to show that males may suffer reductions in relative brain size for the development and maintenance of elaborate sexual weapons.

Artiodactyl species experiencing more intense sexual selection have greater sexual dimorphism in body size suggesting sexual contests may be the leading force driving differences between male and female morphology (Cassini, 2020). Sexual selection apparently acts so strongly on males to invest in progressively larger weapons that it creates inequity in the brain sizes of males and females, with brain size of males decreasing relative to conspecific females in species with the largest weapons (Fig. 1C). Since EQ has sometimes been used as a rough estimate of cognitive ability in animals (Kotrschal et al., 2015; but see also van Schaik et al. 2021, Roth and Dicke 2017; Reader & Laland, 2002; Sol et al., 2005b; Stankowich & Romero, 2017), this suggests that males investing relatively less in brain size compared to females may suffer detrimental effects on cognitive and innovative ability.

Mammalian teeth scale isometrically with body size (Creighton, 1980), and, within tragulids, the cranium and mandible scale at similar rates among males and females, but males have higher upper canine growth rates than females (Terai et al., 1998). Tusks – enlarged male canines – appear to be the first sexual weapon to evolve in artiodactyls (Cabrera & Stankowich, 2018); and female canines are relatively smaller than those of their conspecific males. Our results further support the positive scaling relationship between weapon size and body size in male tusked deer, but when compared to females, who lack sexual weapons, relative brain size is larger in conspecific females suggesting a potential reduction in expensive structures within these tusk bearing species. Bovids are unusual among ungulates because females of many species also develop sizeable horns. Bovid females use their horns either for defense against predators or to guard territories against conspecifics (Stankowich & Caro, 2009), so females that invest heavily in horns may also face tradeoffs with relative brain size. We found patterns of sexual dimorphism in relative brain size consistent with this tradeoff, as species with strong sexual dimorphism in weapon size also had the largest difference between male and female brain sizes. The potential difference in energy investment when developing weapons (permanent vs. deciduous) may explain why cervids suffer a reduction with relative brain size. Cervids may invest more in antler production during their early years resulting in a stronger trade-off with other developing organs like the brain. Further analyses are recommended to make stronger inferences about trends during ontogenetic development as our study only focused on adult measurements.

Initially, we hypothesized that, within males, as relative investment in sexual weapons increased, the relative investment in brain size would drop, we found no relationship between relative brain and weapon size in females or males. Although many other studies found little to no support for male costs at larger weapon sizes (Dinh, 2022; McCullough & Emlen, 2013; Somjee, 2021; Somjee et al., 2018) suggesting some weapons might be costly to grow, but not maintain or weapons are not equally costly across every stage of development. We suggest two *post hoc* hypotheses that may explain why males do not appear to pay for their relatively longer weapons with reductions in relative brain size.

First, previous research found horned rhino beetles (*Trypoxylus dichotomus*) suffered no direct fitness tradeoff with immune system, growth of other structures, and overall survival, which may be due to support through neighboring structures (i.e., legs/wings) (McCullough & Emlen, 2013). Biomechanically, horns and antlers are large, weight-bearing cranial weapons which likely require larger, more domed platforms to support the size and weight of these sexual weapons and to withstand the physical stresses of aggressive combat (e.g., torque and impact), possibly imposing strong positive selection for larger cranium size. If large, robust crania are required to support large horns and antlers and if cranium size scales isometrically with weapon size, it is likely that endocranial volume may also scale isometrically, which could explain why our measure of relative brain size (estimated from endocranial volume) did not decline with weapon lengths.

Second, the evolution of larger weapons allows for more extensive pre-combat signaling of fighting ability, especially in cervids and bovids that have large bodies, large weapons, and live in more open habitats where assessment of rivals from a distance is greater (Cabrera & Stankowich, 2018; Emlen, 2008; Geist, 1998; Lopez & Stankowich, 2023). Increased signaling and assessment may require greater cognitive and decision-making abilities in these species, strengthening selection for larger brains. In contrast, tusked species tend to be “slinkers” that tend to be smaller in size, live in more closed habitats, and engage in quick slashing and stabbing combat in close quarters when they meet, without much signaling. In support of this, we found that tusked species, had lower EQ values in both males and females.

While some argue that EQ is a suboptimal measure of cognitive ability (Deaner et al., 2007; van Schaik et al., 2021), it commonly used in large studies of comparative cognition (Boddy et al., 2012; Jerison, 1973; Marino, 1998; Stankowich & Romero, 2017; Tsuboi et al., 2018) because endocranial volume is quickly measured from skulls in museum collections, allowing for a larger sample size and broader taxonomic sampling. Here, we use EQs to examine the relationship between relative brain size and sexual weapon size, rather than as a measure of higher cognition; though the declines in relative brain sizes we found with greater weapon sizes in males relative to females may extend to cognitive effects as well. Future studies should further question if males with larger sexual weapons may energetically compensate with reductions in cranial thickness, musculature, fecundity, or longevity, or with significant increases in energetic intake relative to females of the same species.

## Figures and Tables

**Figure 1:** A) phylogenetic tree of the species (N=29) analyzed in our study. Yellow=Tusks; Black: Both Antlers and Tusks; Blue: Antlers; Pink: Horns. Artwork by Tayyab Qureshi. B) PGLS insignificant association between weapon size and body size in males (skull length as a measure of body size). C) PGLS insignificant association between weapon size and body size in females (skull length as a measure of body size). D) PGLS negative association between male-female relative brain investment (ΔEQ) and male-female relative weapon investment (ΔWQ; Yellow=Tusks; Blue: Antlers; Pink: Horns). *Rangifer tarandus was added postproduction for Fig. 1A, but species is included in Fig. 1B-D.

**Table 1.** Summary of three weapon groups: Horns, Antlers, and Tusks. *Indicates species that bear both tusks and antlers.

Weapon Type	N	Species
Antlers	13	Alces alces, Axis porcinus, Capreolus capreolus, Cervus elaphus, Dama dama, Mazama americana, Odocoileus hemionus, Odocoileus virginianus, Pudu mephistophiles, Muntiacus muntjak*, Muntiacus reevesi*, Elaphodus cephalophus*, Rangifer tarandus
Horns	11	*Antidorcas marsupialis, Capra hircus, Connochaetes taurinus, Damaliscus lunatus, Kobus kob, Litocranius walleri, Nanger granti, Oreotragus oreotragus, Ovis aries, Ovis canadensis, Redunca redunca*
Tusks	8	Elaphodus cephalophus*, Hydropotes inermis, Moschiola meminna, Moschus moschiferus, Muntiacus muntjak*, Muntiacus reevesi*,Tragulus kanchil, Tragulus napu

**Table 2:** This table summarizes results from the uncorrected (‘lm’) and phylogenetically corrected (‘pgls’) log-based regressions. Subsequential calculations to generate EQ and WQ reflect the phylogenetically corrected outputs; **Bold**=significant (p<0.05)

Uncorrected (‘lm’, *N=29)*					
Y	X	β (95%CI)	Intercept	*p*	λ
Log_10_ (Brain Mass)	Log_10_ (Skull Length^3^)	**0.667 (0.603, 0.731)**	−0.612	**<0.001**	NA
Log_10_ (Weapon Length)	Log_10_ (Skull Length)	**2.520 (1.967, 3.073)**	−2.155	**<0.001**	NA
Corrected (‘pgls’, *N=29)*					
Log_10_ (Brain Mass)	Log_10_ (Skull Length^3^)	**0.592 (0.517, 0.668)**	−0.419	**<0.001**	0.859
Log_10_ (Weapon Length)	Log_10_ (Skull Length)	**2.091 (1.290, 2.889)**	−1.732	**<0.001**	0.718

**Table 3:** This table summarizes results from sex specific (M, F) and sexual dimorphic (M-F) PGLS analyses testing the relationship between relative weapon size and relative brain size. **Bold**=significant (p<0.05). Weapon type (Horns, Tusks, or Antlers) was included as a factor in these models; Antlers serve as the reference value in our regression models.

*N=29*; ‘pgls’		β	*p*	λ
M EQ	M WQ	0.016	0.609	0.000
	*Horns vs. Antlers*	***−0.165***	***0.037***	
	*Tusks vs. Antlers*	***−0.609***	***0.001***	
F EQ	F WQ	0.057	0.417	0.000
	*Horns vs. Antlers*	*−0.126*	*0.248*	
	*Tusks vs. Antlers*	***−0.596***	***0.001***	
M-F EQ	M-F WQ	**−0.073**	**0.014**	0.000
	*Horns vs. Antlers*	***−0.112***	***0.049***	
	*Tusks vs. Antlers*	*−0.129*	*0.093*^	

## Significance Statement:

We took measurements of antlers, horns, and tusks from skulls of both males and females, as well as brain volume and looked at the relationship between relative weapon size and relative brain size. Our work found support that bearing large, exaggerated sexually selected weapons results in a negative relationship with relative brain size: when males invest more in sexual weaponry, they evolve smaller brains relative to females, who typically don’t invest in weaponry. Given that most studies largely are focused on tradeoffs solely within one sex, our study provides a novel approach comparing the relationship between sexes to measure sexual dimorphic investment. The evolution of weaponry in ruminants is one of the most widely studied topics of the last 70 years and this study yields new support for the possible presence of sexual dimorphic trade-offs amongst sexually selected traits.
